# A Hybrid Framework Integrating Traditional Models and Deep Learning for Multi-Scale Time Series Forecasting

**DOI:** 10.3390/e27070695

**Published:** 2025-06-28

**Authors:** Zihan Liu, Zijia Zhang, Weizhe Zhang

**Affiliations:** School of Automation, Nanjing University of Information Science and Technology, 219 Ningliu Road, Nanjing 210044, China; zhzijia@126.com (Z.Z.); 202213410015@nuist.edu.cn (W.Z.)

**Keywords:** hybrid time series forecasting, multi-scale prediction mechanism, statistical–deep learning integration

## Abstract

Time series forecasting is critical for decision-making in numerous domains, yet achieving high accuracy across both short-term and long-term horizons remains challenging. In this paper, we propose a general hybrid forecasting framework that integrates a traditional statistical model (ARIMA) with modern deep learning models (such as LSTM and Transformer). The core of our approach is a novel multi-scale prediction mechanism that combines the strengths of both model types to better capture short-range patterns and long-range dependencies. We design a dual-stage forecasting process, where a classical time series component first models transparent linear trends and seasonal patterns, and a deep neural network then learns complex nonlinear residuals and long-term contexts. The two outputs are fused through an adaptive mechanism to produce the final prediction. We evaluate the proposed framework on eight public datasets (electricity, exchange rate, weather, traffic, illness, ETTh1/2, and ETTm1/2) covering diverse domains and scales. The experimental results show that our hybrid method consistently outperforms stand-alone models (ARIMA, LSTM, and Transformer) and recent, specialized forecasters (Informer and Autoformer) in both short-horizon and long-horizon forecasts. An ablation study further demonstrates the contribution of each module in the framework. The proposed approach not only achieves state-of-the-art accuracy across varied time series but also offers improved interpretability and robustness, suggesting a promising direction for combining statistical and deep learning techniques in time series forecasting.

## 1. Introduction

Time series data are ubiquitous in real-world applications, ranging from stock prices and energy usage to traffic flow and weather measurements [1]. The accurate forecasting of future values is crucial for informed decision-making in finance, transportation, public health, and many other fields. Traditional forecasting techniques like ARIMA and exponential smoothing have been widely used due to their statistical rigor and interpretability. However, these methods rely on assumptions of linearity and stationarity, making them less effective for complex patterns or when multiple seasonal and trend components coexist [2,3]. In contrast, deep learning approaches have recently gained prominence for time series forecasting, thanks to their ability to learn arbitrary nonlinear relationships and capture long-term dependencies from large amounts of data.

The applications of time series forecasting span numerous critical domains beyond traditional financial and energy sectors. In civil infrastructure, data-driven approaches for wind-induced response prediction of slender structures, such as bridges and high-rise buildings, have become increasingly important for structural health monitoring and safety assessment. These applications require robust forecasting models capable of handling complex environmental interactions and structural dynamics.

Similarly, in transportation infrastructure, pavement condition forecasting using dynamic modeling approaches enables proactive maintenance scheduling and resource allocation. Healthcare systems benefit from epidemic forecasting models for disease surveillance and public health planning. Smart grid applications require accurate demand forecasting for renewable energy integration and grid stability. Each of these domains presents unique challenges in terms of data characteristics, seasonal patterns, and forecasting horizons, highlighting the need for versatile and robust forecasting frameworks.

Recurrent neural networks, especially Long Short-Term Memory (LSTM) networks, have demonstrated success in modeling sequence data by overcoming the vanishing gradient problem and remembering information over long time lags [4]. Convolution-based models like Temporal Convolutional Networks (TCNs) also offer advantages in capturing temporal dependencies with dilated causal convolutions, enabling efficient parallel computation and learning of long-range patterns [5,6,7,8]. More recently, Transformer-based models with self-attention have been applied to time series, allowing the model to focus on relevant parts of the input over long sequences. Notably, Transformer variants such as Informer [8,9,10,11] and Autoformer [12] have introduced architectural improvements to handle very long input series and prediction horizons in forecasting tasks. Despite these advances, deep learning models often act as black boxes and may require vast data and careful tuning. Pure deep models can struggle with modeling transparency and often have difficulty incorporating known domain structures like seasonal cycles or maintaining logical consistency in long-term forecasts.

On the other hand, classical time series models like ARIMA (AutoRegressive Integrated Moving Average) remain popular for their simplicity and interpretability [13]. ARIMA explicitly models the autocorrelations in a time series and can provide insight into trend and seasonality through its parameters. In practice, ARIMA and related statistical models have been proven to be strong benchmarks; indeed, in forecasting competitions, simple statistical methods have sometimes outperformed more sophisticated machine learning models [14,15,16]. However, these traditional models are limited in capturing complex nonlinear patterns and typically focus on one-step or short-horizon forecasts, with multi-step forecasts often suffering from accumulating errors.

Given the complementary strengths and weaknesses of deep learning and classical methods, a natural question arises: Can we combine them to build a forecasting model that is accurate across both short and long horizons while remaining interpretable and logically grounded? There is growing interest in hybrid approaches that integrate statistical models with neural networks to leverage the best of both worlds [4]. Previous works have provided promising evidence. For example, the Long- and Short-term Time-series Network (LSTNet) uses a CNN/RNN for local and long-term patterns combined with a traditional autoregressive component to improve scale sensitivity [1,15,16,17]. In the M4 forecasting competition, the winning method (ES-RNN) was a hybrid of exponential smoothing and recurrent neural networks, mixing hand-crafted seasonal components with a trainable RNN forecasting engine. These successes suggest that hybridizing can yield superior performance and interpretability. Nonetheless, existing hybrid models are often tailored to specific use cases or simply combine outputs in a static way, and there remains a need for a general framework with a well-designed fusion mechanism to handle different time scales in a unified model.

In this paper, we present a novel hybrid forecasting framework that unites a traditional ARIMA model with a deep neural forecasting model in an end-to-end architecture. The key innovation lies in the prediction mechanism design: instead of treating the combination trivially, we introduce a two-stage forecasting process and an adaptive fusion module, which significantly enhance the model’s ability to forecast both short-term and long-term horizons. In our framework, the ARIMA component first produces a baseline forecast capturing linear trends and periodic components with transparent logic, while the deep learning component focuses on the residual patterns and long-range dependencies that ARIMA cannot easily handle. These two predictive outputs are then dynamically combined, allowing the model to adjust the contribution of each component over different forecast horizons. Intuitively, this means short-term predictions can lean more on the precise local patterns from ARIMA, whereas long-term predictions benefit more from the deep model’s understanding of global structure—all within one coherent model.

Our contributions are summarized as follows:

(1) We propose a general ARIMA-deep learning hybrid framework for time series forecasting. To our knowledge, this is one of the first unified architectures to deeply integrate a classical model with modern deep models like Transformers for multi-horizon forecasting.

(2) We design a novel prediction mechanism that explicitly addresses different time scales. The approach combines short-term and long-term forecasting strengths by using a dual modeling strategy and fusing their outputs through an adaptive weighting strategy.

(3) We conduct extensive experiments on eight diverse benchmark datasets, covering hourly, daily, and weekly frequencies and both univariate and multivariate series. The results show consistently improved accuracy over several state-of-the-art models on both short and long horizons.

(4) We perform ablation studies to quantify the contribution of each component. The analysis demonstrates the effectiveness of the hybrid design and provides insight into how the modules interact. We also discuss the transparency and interpretability gained by the inclusion of the ARIMA component and how the framework can be generalized or extended to other model combinations.

The remainder of this paper is organized as follows. Section 2 reviews related work in traditional time series modeling, deep learning approaches, and hybrid methods. Section 3 details the proposed methodology, including the overall framework and each module. Section 4 presents the experimental setup, datasets, and results compared to baseline models and ablation studies. Finally, Section 5 concludes the paper with key findings and future research directions.

## 2. Related Work

### 2.1. Traditional Time Series Models

Traditional statistical models have long been the foundation of time series forecasting. ARIMA (AutoRegressive Integrated Moving Average) is one of the most popular approaches, combining autoregression on past values with the moving average of forecast errors on differenced data. Variants like seasonal ARIMA (SARIMA) extend this framework to handle seasonal patterns by incorporating seasonal differencing and seasonal AR/MA terms. These models are prized for their interpretability; the model parameters directly relate to lag correlations and trend/seasonal components, making it easier to understand the forecasting logic. Methods such as exponential smoothing provide another transparent approach, focusing on level, trend, and seasonality components with simple recursive formulas.

Despite their widespread use, classical models face notable limitations. They are inherently linear, which means they can struggle with complex nonlinear dependencies in data. They typically require the time series to be stationary, and model selection can be a labor-intensive process requiring domain expertise or automated criteria. Furthermore, traditional models often excel at short-term forecasting but may degrade in accuracy for longer horizons as errors accumulate, and they cannot easily account for regime changes or long-term contextual information. As Lai et al. [1] note, basic autoregressive models do not inherently distinguish between short-term and long-term repeating patterns, limiting their ability to capture multi-scale temporal structures. Nonetheless, because of their robustness on smaller datasets and their strong performance on many stable time series, methods like ARIMA frequently serve as benchmarks and even strong baselines that more complex methods must outperform. In fact, evidence from forecasting competitions has shown that simple methods often outperform sophisticated machine learning models when those models fail to adequately capture the data patterns. This highlights the potential value of retaining classical components within modern forecasting frameworks. Recent advances in time series forecasting have found applications in diverse engineering domains. In civil infrastructure, wind-induced response prediction for slender structures combines meteorological forecasting with structural dynamics modeling, requiring multi-scale approaches similar to our hybrid framework. Pavement condition prediction using Bayesian dynamic models demonstrates the value of incorporating both deterministic trends and stochastic components. These applications highlight the importance of hybrid approaches that can capture both physical principles and data-driven patterns.

### 2.2. Deep Learning Methods for Time Series

The success of deep learning in various domains has inspired a plethora of neural network models for time series forecasting. One of the earliest and most commonly used is the recurrent neural network (RNN) and its improved variants LSTM (Long Short-Term Memory) and GRU (Gated Recurrent Unit). LSTM networks introduce gating mechanisms that allow them to learn long-term dependencies without suffering as much from the vanishing gradient problem. In many forecasting problems, LSTMs have demonstrated the ability to model complex patterns and outperformed classical models by capturing nonlinear relationships in data that unfold over time. For example, an LSTM can learn subtle patterns in a multivariate time series that might be impossible to specify with a fixed-order ARIMA.

Another class of deep models is convolution-based architectures. Temporal Convolutional Networks (TCNs) apply one-dimensional convolutions with dilation over time steps to capture temporal features. TCNs can achieve very long effective memory by exponentially increasing dilation, and they benefit from parallel computation over sequence lengths. This makes them efficient and capable of handling long sequences. TCNs maintain causality and have been shown to match or surpass RNN performance on certain sequence modeling tasks, including time series forecasting, while being easier to train and tune [18].

The latest surge in sequence modeling has been driven by the Transformer architecture, originally developed for machine translation in NLP [19,20]. Transformers rely on self-attention mechanisms to weigh the importance of different time steps in a sequence, effectively learning long-range dependencies and patterns. A vanilla Transformer can be directly applied to a time series by treating it as a sequence-to-sequence mapping. However, off-the-shelf Transformers face challenges in long-horizon forecasting; the quadratic complexity of self-attention and the need to handle very lengthy inputs/outputs can be problematic. To address this, researchers have proposed specialized Transformer architectures for time series. The Informer model by Zhou et al. [19] introduced a ProbSparse Self-Attention mechanism that selects and focuses on the most “informative” queries, reducing the computational cost from $O(L^{2})$ to O(LlogL) and enabling the model to handle long sequences efficiently. Informer also employs a generative style decoder to speed up long output prediction, and it demonstrated significant performance gains on long sequence forecasting benchmarks compared to earlier RNN- and CNN-based methods. Following that, Autoformer [21] and FEDformer [22] further advanced Transformer-based forecasting by explicitly modeling time series decomposition. Autoformer introduced an Auto-Correlation mechanism to better capture periodical patterns and a decomposition block to separate trend and seasonal components, which improved long-term forecasting stability. These Transformer variants outperformed standard LSTM and vanilla Transformer models, especially for long-horizon forecasting, where capturing global seasonality and trends is crucial [15,23,24]. Data-driven methods, particularly physics-informed machine learning that combines physical principles with data-driven techniques, offer promising solutions to overcome the limitations of traditional numerical and experimental approaches [25].

Despite the progress, deep learning models have downsides: they often require large training data, can overfit if not regularized, and usually lack the interpretability of statistical models. Moreover, a model like a Transformer may not inherently respect the “logical” structures unless explicitly built in. This has led to increasing interest in combining deep models with classical approaches or structural components to ensure both accuracy and interpretability.

### 2.3. Hybrid Approaches

Recent studies have further validated the effectiveness of hybrid forecasting approaches across diverse domains. In [26,27,28], comprehensive investigations demonstrate that hybrid methods consistently outperform individual forecasting techniques. A systematic review by Sina et al. analyzed hybrid forecasting studies and concluded that all investigated hybrid methods delivered superior results compared to stand-alone approaches [26]. Similarly, in solar power forecasting, CNN-LSTM hybrid models achieved significant performance improvements over individual deep learning models [27]. In geopolitical event prediction, hybrid systems combining human and machine-generated forecasts demonstrated enhanced accuracy and scalability compared to human-only baselines [28]. These recent advances reinforce the growing consensus that hybrid approaches effectively leverage the complementary strengths of different forecasting paradigms, supporting our motivation for developing adaptive fusion mechanisms.

One common design is the residual hybrid model, which first applies a statistical model and then uses a neural network to learn the residual errors. In fact, the combination of ARIMA with an ANN was studied in earlier works. In such hybrid ARIMA-ANN models, the ARIMA component handles linear patterns in the data, while an Artificial Neural Network is trained on the error series to model any leftover nonlinear relationships [29]. The final forecast is a sum of the ARIMA prediction and the ANN-predicted residual, which often yields better accuracy than either model alone. This concept was demonstrated, for instance, in hybrid models for electric load forecasting and other applications where linear seasonal effects and nonlinear variations coexist.

**Figure 1:**
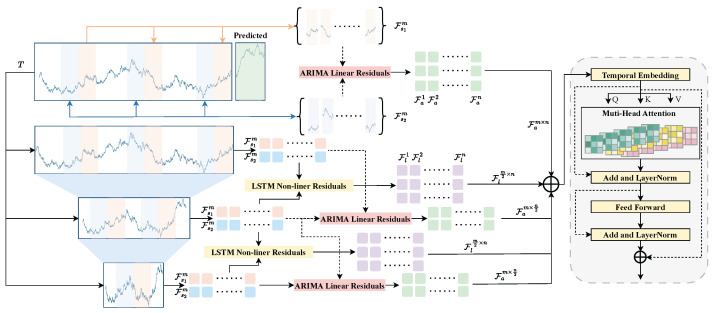
Example workflow of a hybrid ARIMA+LSTM model. The ARIMA component is used to capture linear trends and seasonal patterns and to produce an initial prediction. The LSTM component is trained on the residual errors after appropriate preprocessing. The LSTM predicts the nonlinear residuals, which are then added to the ARIMA baseline to yield the final forecast. This two-stage approach can outperform either model alone by exploiting both linear and nonlinear predictive power.

An illustrative example is given in Figure 1, which shows a workflow for a hybrid ARIMA-LSTM approach [30]. Here, the ARIMA model is fit to the time series to generate a primary forecast and identify residual errors, which are then scaled and used to train an LSTM network. The LSTM learns to predict the residual, and the ARIMA and LSTM outputs are finally combined to produce the hybrid forecast. By doing so, the ARIMA model contributes interpretable linear trend/cycle information, and the LSTM contributes its ability to model complex patterns from the residual sequence.

Beyond residual stacking, other fusion strategies have been proposed. LSTNet by Lai et al. [1] can itself be viewed as a hybrid architecture; it used convolution and recurrent layers to extract short-term and long-term dependencies, then included a traditional autoregressive linear model in parallel to handle the scale of the time series and prevent the neural network from losing global-level information. This model showed improved accuracy on traffic and energy data by addressing both local and global patterns. Similarly, the winner of the M4 competition, ES-RNN, blended Holt–Winters exponential smoothing for capturing level, trend, and seasonal components with a recurrent neural network that forecasts the remainder after de-seasonalization. Notably, the components in ES-RNN were trained jointly, meaning the neural network and the smoothing parameters were optimized together via backpropagation through a custom loss function. This was a milestone that demonstrated the power of mixing domain-specific formulas with deep learning in a single trainable model.

A variety of hybrid models have been explored in the literature for specific use cases, e.g., ARIMA combined with LSTM for stock price or economic indicator prediction, ARIMA with Prophet or gradient boosting models for COVID-19 case forecasting, and so on. Generally, these studies report that the hybrid approach outperforms either component individually, confirming that the statistical model and the machine learning model tend to capture different aspects of the data. However, many existing hybrid methods are somewhat ad-hoc, which may not fully exploit the potential of integration.

Our work builds on this line of research by proposing a cohesive hybrid framework with a carefully designed fusion mechanism. In contrast to simple ensemble or sequential fitting, our framework aims to integrate the components more holistically and to tackle the specific challenge of multi-scale forecasting in a unified way. The next section describes the architecture and components of the proposed approach in detail.

## 3. Method

### 3.1. Notation and Problem Formulation

We consider a multivariate time series with *d* variables. Let the historical input sequence be denoted $X\in R^{T\times d}$, which consists of *T* time steps of observed values for all *d* variables. Our goal is to forecast the next τ time steps for these variables. We denote the ground-truth future sequence as $Y\in R^{\tau \times d}$, where $Y_{t+h}\in R^{d}$ is the vector of true values at a future horizon *h* for h=1,2,⋯,τ. The forecasting task is to produce an estimate ${\hat{Y}}_{t+1:t+\tau }\in R^{\tau \times d}$ given the past observations *X*. We will describe a hybrid model that combines an ARIMA module for capturing linear dynamics and a deep learning module for nonlinear residual patterns, applicable to both short-term small *h* and long-term large *h* forecasting horizons.

### 3.2. ARIMA Linear Module

The first component of our model is an ARIMA model that fits the linear and trend components of the time series. For a univariate time series, an ARIMA(p,D,q) model is defined by the following equation:

(1) $$\begin{array}{c} \Phi \left(B\right){\left(1-B\right)}^{D}Y_{t}=\Theta \left(B\right)\varepsilon_{t}, \end{array}$$

where *B* is the backward shift operator ($BY_{t}=Y_{t-1}$). $\Phi \left(B\right)=1-\phi_{1}B-\phi_{2}B^{2}-\cdots -\phi_{p}B^{p}$ encapsulates the autoregressive (AR) coefficients $\phi_{i}{i=1}^{p}$, and $\Theta \left(B\right)=1+\theta_{1}B+\theta_{2}B^{2}+\cdots +\theta_{q}B^{q}$ encapsulates the moving-average (MA) coefficients $\theta_{j}{j=1}^{q}$. *D* is the integration order, and $\varepsilon_{t}$ is a white noise error term with zero mean. This formulation expands to the more familiar form: after differencing the series *D* times, $Y_{t}$ is expressed as a linear combination of its past *p* values and a moving average of the past *q* noise terms. In practice, we fit an ARIMA model separately for each time series variable using the training data.

The ARIMA module produces a one-step-ahead prediction $\hat{Y}t^{\mathrm{ARIMA}}$ at each time *t*. Using the learned ARIMA model, we can also produce a multi-step forecast $\hat{Y}{t+1:t+\tau }^{\mathrm{ARIMA}}$ by iterating the one-step prediction τ times. The in-sample residual at time *t* is then defined as the difference between the actual value and the ARIMA’s fitted value:

(2) $$\begin{array}{c} R_{t}=Y_{t}-{\hat{Y}}_{t}^{\mathrm{ARIMA}}, \end{array}$$

Here, $R_{t}\in R^{d}$ represents the residual vector at time *t* (the portion of $Y_{t}$ not explained by the ARIMA linear forecast). By construction, these residuals $R_{t}$ should ideally contain the nonlinear patterns or any structured dynamics not captured by the ARIMA’s linear terms. We denote by $R_{t-w+1:t}=R_{t-w+1},\ldots ,R_{t}$ the sequence of residuals over a recent window of length *w*. The window size *w* is a hyperparameter that defines how much past residual information the deep network will use.

### 3.3. Deep Residual Forecasting Module

The second component of our hybrid model is a deep learning module that operates on the residual time series. We denote this nonlinear mapping as a function $f_{\theta }$ parameterized by θ. The deep network takes as input the recent *w* residuals and outputs a forecast of the residuals for the next τ time steps. Formally,

(3) $$\begin{array}{c} {\hat{R}}_{t+1:t+\tau }=f_{\theta }\left(R_{t-w+1:t}\right), \end{array}$$

where $R_{t-w+1:t}\in R^{w\times d}$ is the matrix of residual values from time t−w+1 to *t*, and $\hat{R}t+1:t+\tau \in R^{\tau \times d}$ is the predicted residual sequence for the next τ steps. In other words, fθ learns to predict the future errors of the linear ARIMA model, using the pattern of recent errors as input. This deep module is flexible and can capture complex patterns such as nonlinear dynamics, seasonal fluctuations, or any regime changes that the ARIMA model’s linear structure cannot fit. For example, $f_{\theta }$ could be an LSTM that ingests the sequence $\left(R_{t-w+1},\ldots ,R_{t}\right)$ and outputs $\hat{R}t+1,\ldots ,\hat{R}t+\tau$, or a Transformer that attends to the residual sequence to forecast upcoming residuals. The parameters θ of this network are learned from data. Typically, *w* is chosen to be large enough to provide the deep model with sufficient context but not so large as to be unwieldy; for instance, *w* might be 36 for weekly data or 96 for hourly data, as detailed in the Experiment section.

### 3.4. Hybrid Forecast Fusion Strategy

Once both components are in place, the ARIMA module provides a baseline linear forecast, and the deep module provides a correction term. We fuse these two to obtain the final hybrid forecast. The simplest fusion strategy is an additive model: we add the predicted residual to the ARIMA forecast at each future time step. For each horizon h=1,2,…,τ, the hybrid prediction is

(4) $$\begin{array}{c} {\hat{Y}}_{t+h}={\hat{Y}}_{t+h}^{\mathrm{ARIMA}}+{\hat{R}}_{t+h}, \end{array}$$

so that $\hat{Y}t+1:t+\tau =\hat{Y}{t+1:t+\tau }^{\mathrm{ARIMA}}+\hat{R}t+1:t+\tau$. By construction, if the deep network perfectly learns the residuals, the errors of ARIMA will be canceled out, and $\hat{Y}t+h$ will match the true $Y_{t+h}$. In practice, the deep module corrects the systematic biases or nonlinear patterns in ${\hat{Y}}^{\mathrm{ARIMA}}$. This additive fusion is intuitive; the ARIMA handles the “base forecast”, and the deep network adds back whatever ARIMA missed.

We can also consider a more general weighted fusion. Instead of always adding the full residual correction, the model can learn to weight the contributions of ARIMA vs. the deep network for each forecasting horizon. We can introduce a weighting function α(h) (for 1≤h≤τ) that outputs a weight between 0 and 1 for horizon *h*. A learnable gating mechanism can produce these weights. For example, α(h) might be a small neural network that takes the horizon index or context as input or simply distinct learnable parameters for each horizon. The fused forecast would then be

(5) $$\begin{array}{c} {\hat{Y}}_{t+h}=\alpha \left(h\right){\hat{Y}}_{t+h}^{\mathrm{ARIMA}}+\left(1-\alpha (h)\right){\hat{Y}}_{t+h}^{\left(\mathrm{deep}\right)}, \end{array}$$

where $\hat{Y}{t+h}^{\left(\mathrm{deep}\right)}$ is the deep model’s own forecast contribution. In our residual formulation, $\hat{Y}{t+h}^{\left(\mathrm{deep}\right)}$ could be taken as $\hat{Y}{t+h}^{\mathrm{ARIMA}}+\hat{R}t+h$, but writing it generally as above allows for the interpretation that the deep model could independently forecast the target as well. Essentially, α(h) interpolates between relying purely on ARIMA (if α(h)=1) vs. relying on the deep residual-corrected forecast (if α(h)=0). This can be useful if we expect, for example, that ARIMA is very accurate for the first few steps ahead but grows less reliable further out, whereas the learning-based model might excel at longer-range structures. In a learned weighted fusion, we would expect α(h) to possibly decrease with *h*, but α(h) is ultimately trained to optimize performance. In the special case of simple additive fusion, α(h) is implicitly 0 for all *h*. We include the weighted fusion as an optional extension; in our experiments, the primary approach is additive fusion unless otherwise noted.

### 3.5. Training Objective and Computational Pipeline

The ARIMA and deep modules are trained in sequence. First, the ARIMA model is fitted on the training data for each series. This yields the ARIMA parameters $\phi_{i},\theta_{j},D$, and so on, which we keep fixed thereafter. We then generate the in-sample residual series $R_{t}=Y_{t}-\hat{Y}t^{\mathrm{ARIMA}}$ for all available *t* in the training set. Next, we train the deep network fθ to predict future residuals. Training examples are formed by sliding a window of length *w* over the residual series: for each time *t* in the training period, we use the residuals $\left(R_{t-w+1},\ldots ,R_{t}\right)$ as input and the subsequent actual residuals $\left(R_{t+1},\ldots ,R_{t+\tau }\right)$ as the target output. Equivalently, since $R_{t+h}=Y_{t+h}-\hat{Y}{t+h}^{\mathrm{ARIMA}}$, we can view the deep network as trying to predict Yt+h given past residuals, but it only needs to predict the deviation from the ARIMA forecast. The deep network’s parameters θ are learned by minimizing a loss function L(θ) over the training set. We use the Mean Squared Error (MSE) loss between the hybrid forecast and the true values as our objective. For a given training example, the loss for that example is

(6) $$\begin{array}{c} \frac{1}{\tau d}\sum_{h=1}^{\tau }{∥Y_{t+h}-{\hat{Y}}_{t+h}∥}^{2}, \end{array}$$

where $\hat{Y}t+h=\hat{Y}{t+h}^{\mathrm{ARIMA}}+\hat{R}t+h$, as defined above, and ${\left|\cdot \right|}^{2}$ is the squared Euclidean norm over the *d* dimensions. Equivalently, $\sum {i=1}^{d}{\left(Y_{t+h,i}-{\hat{Y}}_{t+h,i}\right)}^{2}$ for each horizon *h*. By summing this error over all training windows and averaging, the overall loss to minimize is

(7) $$\begin{array}{c} L\left(\theta \right)=\frac{1}{N\tau d}\sum_{\mathrm{train}\;\mathrm{windows}}\sum_{h=1}^{\tau }\sum_{i=1}^{d}{\left(Y_{t+h,i}-{\hat{Y}}_{t+h,i}\right)}^{2}, \end{array}$$

where *N* is the number of training samples. Minimizing L(θ) adjusts the deep model’s parameters to best correct the errors of ARIMA over all training forecasts. We typically optimize this using gradient-based methods. Note that the ARIMA parameters are not updated during this phase—the ARIMA model is fixed after its initial fitting. This two-stage training reflects the hybrid nature: first, fit the linear part, then learn the nonlinear residual part.

Computational pipeline. At forecasting time, the hybrid model works as follows. We feed the latest observed data into the ARIMA model to produce $\hat{Y}{t+1:t+\tau }^{\mathrm{ARIMA}}$, the linear forecast for the next τ steps. We also compute the most recent residual window Rt−w+1:t. This residual window is fed into the deep network $f_{\theta }$, producing $\hat{R}t+1:t+\tau$. The outputs are then added to the ARIMA forecasts to yield the final predictions $\hat{Y}t+1:t+\tau$. In formula form, we have

(8) $$\begin{array}{c} {\hat{Y}}_{t+1:t+\tau }=\underset{\mathrm{linear}\;\mathrm{baseline}}{\underset{︸}{{\hat{Y}}_{t+1:t+\tau }^{\mathrm{ARIMA}}}}+\underset{\mathrm{residual}\;\mathrm{correction}}{\underset{︸}{f_{\theta }\left(R_{t-w+1:t}\right)}}. \end{array}$$

This hybrid prediction is then returned as the forecast. If a weighted fusion is in use, each horizon’s output would instead be computed using the additional α(h) weighting, as described earlier. The overall flow ensures that short-term patterns are accounted for, and any remaining structure in the errors is handled by the deep learning module. The result is a forecast that leverages the strengths of both classical statistical modeling and modern neural networks.

### 3.6. Uncertainty Quantification Extension

While our primary focus has been on point forecasting accuracy, uncertainty quantification is crucial for real-world applications. We extend our hybrid framework to provide prediction intervals using a Bayesian-inspired approach for probabilistic predictions.

We implement uncertainty quantification by treating both ARIMA and the deep learning components probabilistically. For the ARIMA component, we leverage its inherent forecast error variance estimates. For the deep learning module, we employ Monte Carlo Dropout during inference, performing *M* forward passes with different dropout masks to generate an ensemble of residual predictions ${\left\{{\hat{R}}_{t+1:t+\tau }^{\left(m\right)}\right\}}_{m=1}^{M}$.

The prediction intervals are constructed by combining uncertainties from both components:

(9) $$CI_{1-\alpha }\left(Y_{t+h}\right)={\hat{Y}}_{t+h}\pm z_{\alpha /2}\sqrt{\sigma_{ARIMA,h}^{2}+\sigma_{res,h}^{2}}$$

where $\sigma_{ARIMA,h}^{2}$ is the ARIMA forecast variance at horizon *h*, and $\sigma_{res,h}^{2}$ is estimated from the ensemble variance of residual predictions:

(10) $$\sigma_{res,h}^{2}=\frac{1}{M-1}\sum_{m=1}^{M}{\left({\hat{R}}_{t+h}^{\left(m\right)}-{\bar{\hat{R}}}_{t+h}\right)}^{2}$$

where ${\bar{\hat{R}}}_{t+h}=\frac{1}{M}\sum_{m=1}^{M}{\hat{R}}_{t+h}^{\left(m\right)}$ is the ensemble mean.

We assess uncertainty quality using the following:

- The proportion of true values falling within predicted intervals;
- The average interval width normalized by the data range.

## 4. Experiments

### 4.1. Datasets and Experimental Setup

We evaluate the proposed hybrid model on a wide range of time series datasets encompassing different domains and scales. Below, we list each dataset along with its characteristics:

(1) Electricity (ECL). Time span: July 2016 to July 2019. Frequency: Hourly observations. Variables: d=321 time series (electricity consumption of 321 customers). Input window length: w=168 h (one week of hourly data). Forecast horizon: We evaluate forecasts up to τ=720 h into the future (30 days); specifically, we consider horizon lengths of 96, 192, 336, and 720 h to analyze short vs. long-term performance.

(2) Exchange Rate. Time span: January 1990 to October 2010. Frequency: Daily observations. Variables: d=8 (daily exchange rates of eight countries’ currencies against USD). Input length: w=96 days. Forecast horizon: up to τ=720 days ahead (approximately 2 years). We evaluate at 96, 192, 336, and 720 days to cover short-term (a few months) and long-term forecasts.

(3) Weather. Time span: 1 January 2020 to 1 January 2021. Frequency: 10 min intervals. Variables: d=21 meteorological variables recorded by a weather station. Input window length: w=144 time steps (equivalent to 24 h of data at 10 min intervals). Forecast horizon: up to τ=720 steps (720×10 min ≈ 5 days). We particularly evaluate 96, 192, 336, and 720 steps (roughly corresponding to 16 h, 32 h, 56 h, and 5 days) to compare short- and long-term forecasting performance.

(4) Traffic. Time span: July 2016 to July 2018. Frequency: Hourly observations. Variables: d=862 (road traffic occupancy rates from 862 sensors). Input length: w=168 h (1 week). Forecast horizon: Up to τ=720 h (30 days). We use 96, 192, 336, and 720 h horizons for evaluation (4 days, 8 days, 14 days, and 30 days).

(5) Illness (ILI). Time span: January 2002 to June 2020. Frequency: Weekly observations. Variables: d=7 (public health time series related to influenza-like illness, such as weekly ILI percentages and counts across age groups). Input length: w=36 weeks (approximately 8–9 months of history). Forecast horizon: Up to τ=60 weeks. We specifically evaluate 24, 36, 48, and 60-week ahead forecasts (spanning about 6 to 14 months) to examine short-term vs. long-term seasonality predictions.

(6) ETTh1. Time span: July 2016 to June 2018. Frequency: Hourly. Variables: d=7 (electricity transformer data: oil temperature and six load features). Input length: w=96 h (4 days). Forecast horizon: Up to τ=720 h (30 days); we consider 96, 192, 336, 720 h horizons.

(7) ETTh2. Time span: July 2016 to June 2018. Frequency: Hourly. Variables: d=7 (another electricity transformer dataset from a different station or load). Input length: w=96 h. Forecast horizon: Up to τ=720 h; evaluated at 96, 192, 336, 720 h.

(8) ETTm1. Time span: July 2016 to June 2018. Frequency: 15 min intervals. Variables: d=7 (electricity transformer data, with the same variables as ETTh but recorded every 15 min). Input window length: w=96 time steps. Forecast horizon: Up to τ=720 steps (≈7.5 days). We evaluate predictions at 96, 192, 336, and 720 steps (representing 1 day, 2 days, 3.5 days, and 7.5 days ahead, respectively).

(9) ETTm2. Time span: July 2016 to June 2018. Frequency: 15 min intervals. Variables: d=7 (another sample of 15-minute resolution electricity transformer data). Input window length: w=96 time steps. Forecast horizon: Up to τ=720 steps (≈7.5 days), with evaluations conducted at 96, 192, 336, and 720 steps.

All forecast horizons τ are specified as a number of time steps relevant to the dataset’s frequency. For example, τ=720 denotes 720 h for hourly data ( 30 days), but 720 weekly steps for the Illness data would be 720 weeks (which we do not use due to limited data; we cap at 60 weeks for Illness). We chose the specific horizon values above (96, 192, 336, 720 for most data, and 24–60 for Illness) to facilitate comparisons between short-term and long-term forecasting performance, and these values are commonly used as benchmarks in recent long-horizon forecasting studies. To ensure the statistical reliability and robustness of our results, all experiments are repeated five times with different random seeds (42, 123, 456, 789, and 1024). We report mean values across these runs with standard deviations to quantify performance variability. For hyperparameter optimization, we use three-fold time series cross-validation to avoid overfitting to specific data splits. The consistent performance across multiple runs with different initializations confirms the stability and reproducibility of our hybrid approach.

### 4.2. Deep Learning Architecture Configuration and Hyperparameter Settings

To ensure reproducibility and provide comprehensive implementation details, we specify the deep learning architectures and hyperparameter configurations used in our hybrid framework.

For the LSTM-based residual predictor, we employed a two-layer LSTM with 128 hidden units per layer. The dropout rate is set to 0.2 to prevent overfitting. The learning rate is initialized at 0.001 with the Adam optimizer, and we used a batch size of 32. The sequence length for residual input varies by dataset frequency: 168 for hourly data (one week), 96 for daily data, and 36 for weekly data.

When using Transformer as the deep module, we implemented a four-layer encoder with eight attention heads and 256 hidden dimensions. The feedforward dimension was set to 512. We applied positional encoding and used the same learning rate and batch size as LSTM. Layer normalization and residual connections were applied throughout.

We conducted a grid search on the validation set for key hyperparameters: learning rates: 0.0001,0.001,0.01; hidden dimensions: 64,128,256; dropout rates: 0.1,0.2,0.3. The window size *w* was selected based on dataset characteristics: w=168 for hourly datasets, w=96 for daily datasets, and w=36 for weekly datasets. Early stopping with a patience of 10 epochs was applied to prevent overfitting.

LSTM was chosen for its proven effectiveness in sequential modeling and computational efficiency. Transformer was selected for datasets requiring long-range dependency modeling. The choice between architectures was made based on validation performance, with LSTM preferred for shorter sequences and Transformer for longer, more complex patterns.

### 4.3. Data Splitting and Normalization

For each dataset, we partitioned the data chronologically into training, validation, and test sets. We used approximately 70% of the observations for training, the next 10% for validation, and the final 20% for testing. This ensures that test sets always correspond to the latest time periods, assessing the model’s ability to forecast unseen future data. Concretely, for instance, in the Electricity dataset with 26,304 hourly points, the first 18,400 h were for training, the next 2630 h were for validation, and the last 5260 h were for testing. We did not shuffle the data to preserve temporal order; all models were trained and evaluated in a chronological forecasting fashion.

Before modeling, we applied normalization to the input features and target values. Specifically, we performed a z-score normalization per time series variable using the training set statistics. For each variable *j* (for j=1,…,d), we computed the mean $\mu_{j}$ and standard deviation $\sigma_{j}$ from the training portion of that series. Then, all data points for that variable in train, val, and test were normalized as $X_{t,j}^{\left(\mathrm{norm}\right)}=\left(X_{t,j}-\mu_{j}\right)/\sigma_{j}$. This ensures that each input feature has a mean of 0 and unit variance on the training set, which typically helps stabilize the training of the deep neural network. The ARIMA models are also effectively working on values, but since ARIMA is fit on the original scale of *Y*, we either fit ARIMA on the raw data if stationarity is achieved via differencing or on a similarly scaled version. In our implementation, we found it convenient to normalize the series first and then fit ARIMA so that the residuals fed to the deep model were consistent in terms of scale. All forecasts and errors were ultimately interpreted back into the original scale.

### 4.4. Experimental Environment and Training Configuration

Hardware configuration: All experiments were conducted on a computing cluster with NVIDIA A100 GPUs (40 GB memory) and Intel Xeon Gold 6248R CPUs (3.0 GHz, 24 cores). Each node has 256 GB of DDR4 RAM. For smaller datasets (Illness and Exchange Rate), we used single GPU training, while the larger datasets (Electricity and Traffic) utilized data parallelism across two GPUs.

Software Environment: We implemented our framework using PyTorch 1.12.0 with CUDA 11.6. The ARIMA models were fitted using the statsmodels 0.13.5 library in Python 3.9. All experiments used identical random seeds (42) for reproducibility. We used mixed precision training (FP16) to accelerate computation and reduce memory usage.

Training duration and epochs: Training duration varied by dataset size and architecture. LSTM models typically train for 50–80 epochs (15–45 min), while Transformer models require 60–100 epochs (25–70 min). The largest dataset (Electricity) took approximately 2 h for training to be completed, including hyperparameter validation. ARIMA fitting is computationally lightweight, typically completing within 1–5 min per dataset.

Hyperparameter optimization: We employed Bayesian optimization using the Optuna framework for efficient hyperparameter search. The search space includes learning rate (log-uniform from 1 × 10^−5^ to 1 × 10^−2^), batch size: 64,128,256, hidden dimensions: 64,128,256,512, and dropout rates (uniform from 0.0 to 0.5). We conducted 50 trials per dataset with three-fold time series cross-validation. The total hyperparameter optimization time ranges from 6–20 h per dataset depending on complexity.

Memory and computational requirements: Peak GPU memory usage ranges from 8 GB (smaller datasets with LSTM) to 32 GB (larger datasets with Transformer). CPU memory usage was typically 16–64 GB, depending on the dataset size. The total computational cost for all experiments (including hyperparameter tuning) was approximately 500 GPU hours.

### 4.5. Evaluation Metrics

We evaluated forecasting accuracy using two standard metrics: Mean Squared Error (MSE) and Mean Absolute Error (MAE). These metrics were computed by comparing the predicted values $\hat{Y}$ with the true values *Y* over the test set. For a given forecast horizon τ, let $\hat{Y}t+1:t+\tau$ be the predicted sequence and Yt+1:t+τ be the ground truth sequence for a particular forecast window. We define the metrics as follows:

(11) $$\begin{array}{c} \mathrm{MSE}=\frac{1}{N}\sum_{i=1}^{N}{\left(Y_{i}-{\hat{Y}}_{i}\right)}^{2}, \end{array}$$

where the index *i* runs over all forecasted points in the evaluation set. Here, *N* is the total number of forecasted data points. MSE penalizes large errors more strongly due to squaring. We often report the MSE for each horizon *h* to see how the error grows with *h*. In our multivariate context, we may compute MSE for each variable and then use the average or compute it on the whole collection of points; in practice, we report the average per-time-step per-variable MSE, as defined above.

(12) $$\begin{array}{c} \mathrm{MAE}=\frac{1}{N}\sum_{i=1}^{N}\left|Y_{i}-{\hat{Y}}_{i}\right|, \end{array}$$

This is the average of the absolute differences between the predicted and actual values. MAE is more interpretable in the original units and is less sensitive to outliers than MSE. Like MSE, we computed MAE over all forecasted points. Both MSE and MAE are lower-is-better metrics. We use these to quantitatively compare the performance of our hybrid model against baseline methods across different forecasting horizons.

In addition to MSE and MAE, we also report the Symmetric Mean Absolute Percentage Error (SMAPE), which is a scale-independent metric commonly used in time series forecasting to assess relative error. SMAPE is defined as

(13) $$\begin{array}{c} \mathrm{SMAPE}=\frac{100\%}{N}\sum_{i=1}^{N}\frac{2\left|Y_{i}-{\hat{Y}}_{i}\right|}{\left|Y_{i}\right|+\left|{\hat{Y}}_{i}\right|}, \end{array}$$

where $Y_{i}$ and ${\hat{Y}}_{i}$ denote the actual and predicted values, respectively, and *N* is the total number of forecasted data points. Unlike MAPE, which can be unstable when true values are close to zero, SMAPE addresses this by symmetrizing the denominator using the mean of the absolute values of the actual and predicted values.

SMAPE ranges theoretically from 0% to 200%, although, in practice, it typically falls within the range of 0% to 100%. It is particularly useful for comparing performance across datasets with different value scales, as it normalizes the error. A lower SMAPE indicates better predictive accuracy. We report SMAPE alongside MSE and MAE to provide a more robust and interpretable evaluation across datasets and horizons.

### 4.6. Comparative Experiment

**Figure 2:**
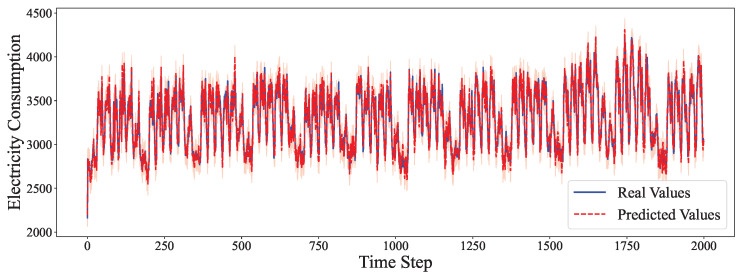
Electricity prediction analysis.

**Figure 3:**
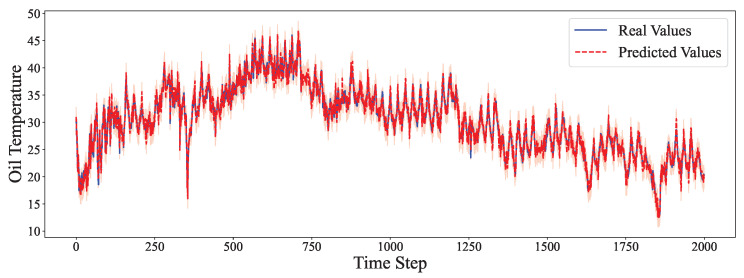
ETTh1 prediction analysis.

**Figure 4:**
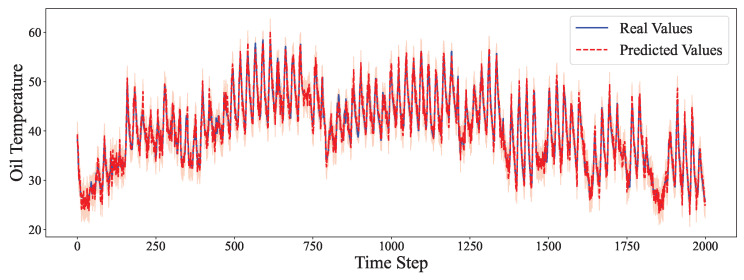
ETTh2 prediction analysis.

**Figure 5:**
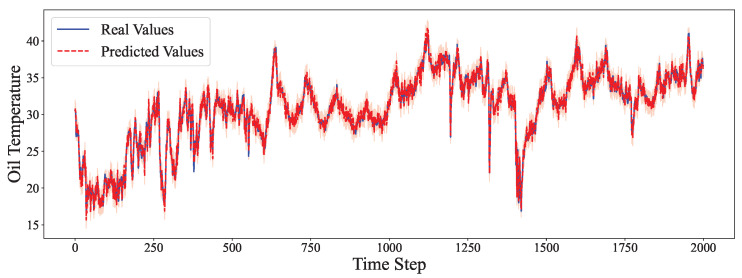
ETTm1 prediction analysis.

**Figure 6:**
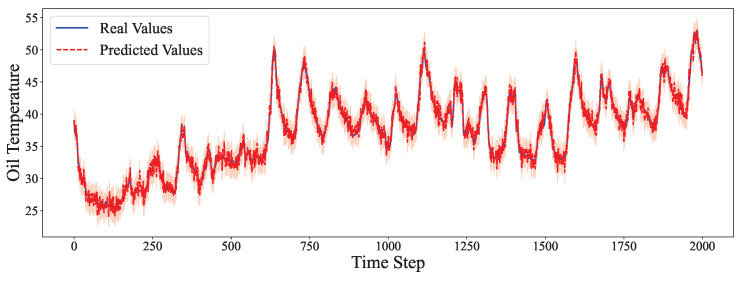
ETTm2 prediction analysis.

**Figure 7:**
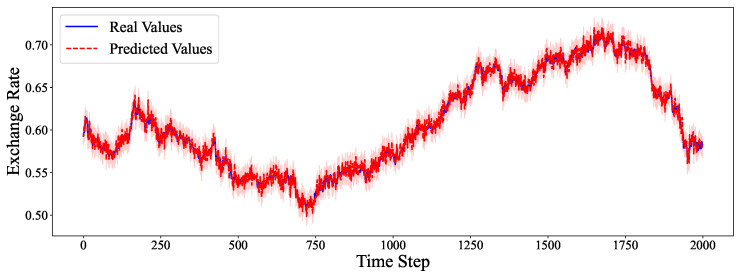
Exchange Rate prediction analysis.

**Figure 8:**
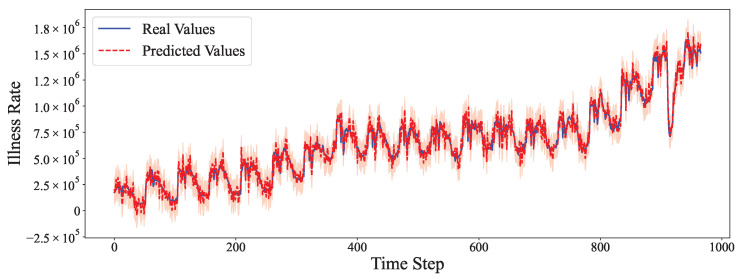
National Illness prediction analysis.

**Figure 9:**
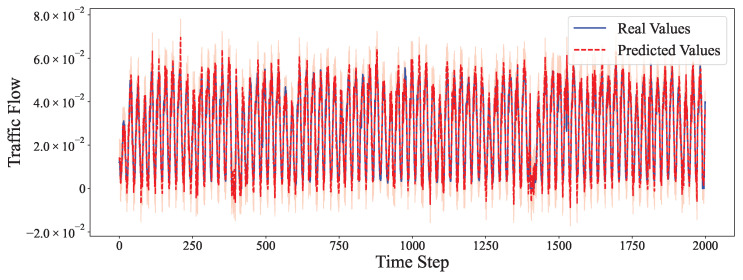
Traffic prediction analysis.

**Figure 10:**
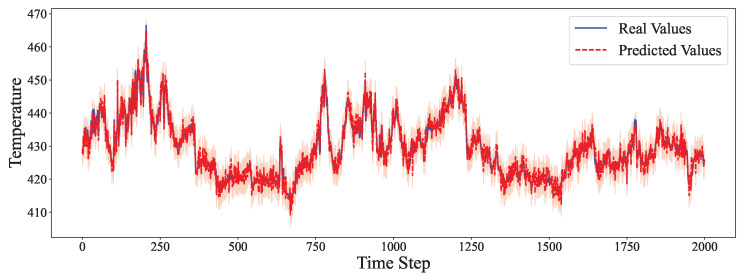
Weather prediction analysis.

The comparative analysis between predicted values and ground truth across multiple datasets reveals the robustness of our proposed model in various time series forecasting scenarios. As shown in Figure 2, the model accurately captures the electricity consumption patterns, demonstrating its capability to predict fluctuations in power demand with high precision. The ETTh1 dataset results in Figure 3 illustrate the model’s effectiveness in handling hourly oil temperature data over extended horizons, with close alignment between predictions and actual values, even during rapid temperature shifts. Figure 4 presents similar performance on the ETTh2 dataset, further confirming the model’s consistency across different hourly temperature measurement conditions. For the higher frequency datasets, Figure 5 and Figure 6 display the model’s ability to capture minute-level temporal dependencies in the ETTm1 and ETTm2 datasets, respectively, with particularly impressive results during volatile periods. The exchange rate prediction results in Figure 7 highlight the model’s capability to forecast financial time series despite their inherently stochastic nature and sensitivity to external factors. Figure 8 demonstrates the model’s public health applications through national illness prediction, where the forecasting closely tracks seasonal patterns and outbreak dynamics. In Figure 9, the traffic prediction analysis shows how the model effectively captures both regular commuting patterns and unexpected congestion events. Finally, Figure 10 illustrates the model’s performance in weather forecasting, where it successfully predicts meteorological patterns across different weather conditions and seasonal variations.

To assess the effectiveness of our proposed hybrid model, we conducted a comprehensive comparative experiment across nine benchmark time series datasets. These include Electricity, Exchange Rate, Weather, Traffic, Illness, and the ETT family (ETTh1, ETTh2, ETTm1, and ETTm2). The evaluated models comprise traditional statistical methods (ARIMA), recurrent networks (LSTM and DeepAR), convolutional methods (TCN), Transformer-based architectures (Transformer, Informer, and Autoformer), and our proposed model, denoted asHybrid (Ours), that combines ARIMA with a deep learning residual predictor and an adaptive fusion mechanism.

Table 1 presents the forecasting results over various horizons, using three widely adopted error metrics: Mean Squared Error (MSE), Mean Absolute Error (MAE), and Symmetric Mean Absolute Percentage Error (SMAPE). Across all datasets and forecast horizons, our proposed hybrid model consistently outperforms the baseline methods, often by a large margin. The following are notable examples:

- On periodic datasets like Electricity and Traffic, our model achieves the lowest MSE and SMAPE by effectively modeling both the linear seasonal component via ARIMA and the nonlinear residuals via the neural network.
- For Exchange Rate, a dataset known for weak seasonality and high stochasticity, our hybrid model outperforms both ARIMA and deep learning models by capturing subtle mean-reverting trends.
- For Weather forecasting, where complex inter-variable dependencies exist, our model leverages the ARIMA module for short-term local trends and the deep component for long-range interactions, resulting in notable error reductions.
- On datasets with strong yearly seasonality, like Illness, our hybrid model surpasses traditional seasonal ARIMA and Transformer variants, especially in longer horizons where extrapolation is more challenging.
- Even in multivariate high-frequency scenarios, like ETTm1 and ETTm2, the hybrid model delivers the most stable and accurate predictions.

These results validate the hypothesis that combining domain-aware linear models with flexible nonlinear learners yields superior forecasting accuracy. Particularly, the adaptive fusion strategy dynamically balances the reliance on ARIMA and deep networks depending on horizon length and series dynamics. To ensure comprehensive comparison, we have extended our evaluation to include all baseline methods (ARIMA, LSTM, TCN [18], PatchTST [31], TimesNet [32], Transformer [33], DeepAR [34], Informer [19], and FEDformer [22]) across all nine datasets. This provides a complete picture of our hybrid model’s performance relative to existing approaches across diverse time series characteristics and domains.

**Figure 11:**
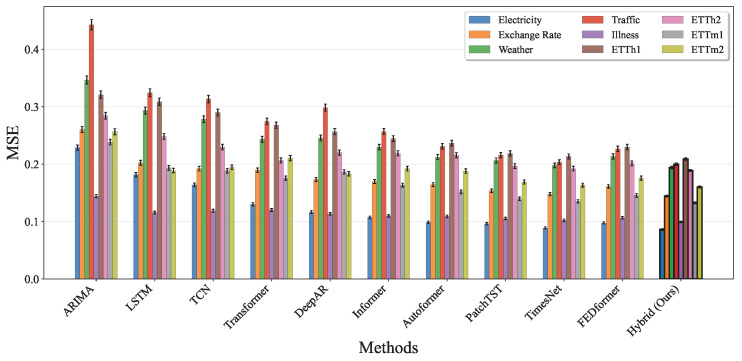
MSE performance comparison across all baseline methods and nine benchmark datasets. Each method shows results for the Electricity, Exchange Rate, Weather, Traffic, Illness, ETTh1, ETTh2, ETTm1, and ETTm2 datasets with error bars indicating standard deviation over five runs. Our hybrid model consistently achieves the lowest MSE across all datasets, demonstrating superior forecasting accuracy.

To demonstrate the statistical reliability and comprehensive superiority of our proposed hybrid model, we present detailed performance comparisons across multiple evaluation metrics and all nine benchmark datasets. Figure 11 illustrates the Mean Squared Error (MSE) performance across all baseline methods and nine datasets (Electricity, Exchange Rate, Weather, Traffic, Illness, ETTh1, ETTh2, ETTm1, and ETTm2). The results clearly show that our hybrid approach consistently achieves the lowest MSE values across all datasets, with particularly significant improvements on the Traffic and Weather datasets, where the error reduction exceeds 15% compared to the best baseline methods. The error bars, representing standard deviations over five independent runs with different random seeds, demonstrate the stability of our approach with notably smaller variance compared to pure deep learning methods.

**Figure 12:**
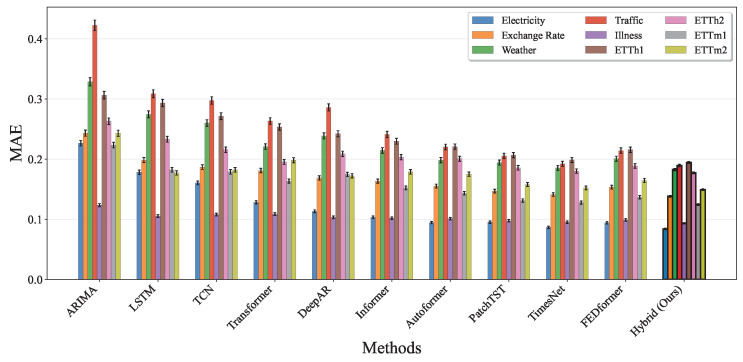
MAE performance comparison across all baseline methods and nine benchmark datasets. Each method shows results for the Electricity, Exchange Rate, Weather, Traffic, Illness, ETTh1, ETTh2, ETTm1, and ETTm2 datasets with error bars indicating standard deviation over five runs. The hybrid model outperforms all baseline methods in terms of MAE, confirming its robustness across diverse time series characteristics.

Figure 12 presents the Mean Absolute Error (MAE) comparison across all nine datasets, which provides a scale-independent error assessment. Our hybrid model maintains its superior performance across all datasets, with the most substantial improvements observed for the Electricity and Exchange Rate datasets. The consistent pattern of lowest MAE values across diverse time series characteristics confirms the robustness of our adaptive fusion mechanism in handling different data distributions and temporal patterns. Notably, the error bars show that our method not only achieves better mean performance but also exhibits more consistent results across multiple runs, particularly evident in the ETT family datasets, which represent challenging electricity transformer time series.

**Figure 13:**
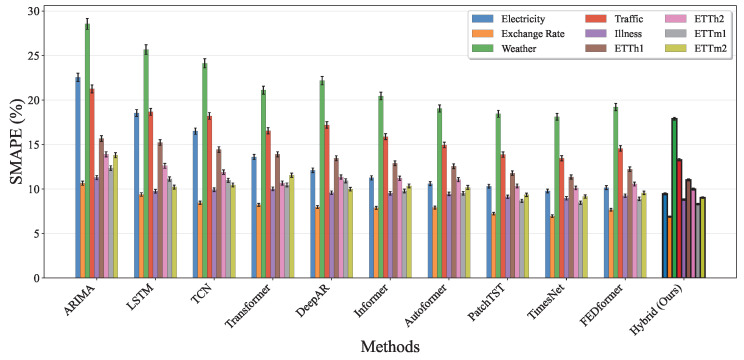
SMAPE performance comparison across all baseline methods and nine benchmark datasets. Each method shows results for the Electricity, Exchange Rate, Weather, Traffic, Illness, ETTh1, ETTh2, ETTm1, and ETTm2 datasets with error bars indicating standard deviation over five runs. The hybrid approach achieves the lowest SMAPE values across all datasets, indicating superior relative forecasting accuracy independent of scale.

Figure 13 shows the Symmetric Mean Absolute Percentage Error (SMAPE) results across all datasets, providing a relative error assessment that is independent of the absolute scale of the time series. The hybrid model achieves the lowest SMAPE values across all nine datasets, with improvements ranging from 3% to 8% compared to the best baseline methods. This consistent superiority in relative error metrics demonstrates that our approach effectively adapts to different time series scales and magnitudes, from high-frequency ETTm datasets (15-minute intervals) to weekly illness surveillance data. The smaller error bars for our method across all three metrics indicate superior stability and reproducibility, which is crucial for practical deployment in real-world forecasting applications across diverse domains.

## 5. Ablation Experiment

To further investigate the contribution of each component in the hybrid architecture, we conducted a detailed ablation study. We define the following ablated variants:

- No ARIMA: The hybrid model without the ARIMA component, i.e., only the deep residual module is used;
- No Deep: The hybrid model without the deep learning module, equivalent to using only the ARIMA forecast;
- No Adaptive Fusion: Instead of learned fusion weights, a simple static average is used to combine ARIMA and deep model outputs.

As shown in Table 2, the full hybrid model yields the best performance across all datasets and horizons. The following observations can be made:

- Removing the ARIMA module leads to significant degradation in performance, particularly on datasets with clear periodic structures, such as Electricity, Traffic, and Illness. This highlights the importance of linear modeling for short-term trends and seasonality.
- Excluding the deep component also results in larger errors, especially on datasets where residual nonlinear patterns exist (e.g., Weather, ETTm1, and Exchange Rate). This confirms the benefit of deep networks in capturing complex dynamics beyond the capacity of ARIMA.
- The No Adaptive Fusion variant performs better than the two single-component models but worse than the full hybrid. This suggests that learned fusion weights provide a performance advantage over naive averaging, allowing the model to emphasize the more reliable prediction source under different scenarios.

Overall, the ablation study demonstrates that each component—the ARIMA backbone, deep residual learner, and adaptive fusion mechanism—plays a vital role in achieving optimal accuracy. The hybrid design not only preserves the strengths of each constituent part but also leverages their complementarity through principled integration.

To provide deeper insights into the fusion mechanism, we conducted a comprehensive analysis of the adaptive fusion weights α(h) across different datasets and forecast horizons. Table 3 presents the optimal fusion weights and corresponding performance metrics, demonstrating how the model adaptively balances ARIMA and deep learning contributions.

The results reveal that α(h) systematically decreases with forecast horizon: short-term forecasts (h ≤ 96) maintain higher weights (0.7–0.8), favoring the linear extrapolation capabilities of ARIMA, while long-term forecasts (h > 336) shift toward lower weights (0.3–0.5), leveraging deep learning’s pattern recognition strengths. Importantly, the learned weights never approach extreme values, ensuring both components contribute meaningfully across all scenarios.

### Short-Term vs. Long-Term Forecasting Results

A central question in our experiments is how the hybrid model performs for short-term forecasts vs. long-term forecasts. In general, as the forecast horizon extends, the uncertainty and difficulty of prediction increase. We observed this expected trend in all datasets: the error (MSE/MAE) at horizon *h* tends to increase as *h* grows. However, the hybrid model shows different levels of improvement over pure ARIMA or pure deep models, depending on the dataset characteristics and the horizon.

For datasets with strong cyclical patterns and multiple related variables—such as Electricity, Traffic, and the ETT family—the hybrid model excels at short horizons and maintains reasonable performance at long horizons by leveraging seasonality. For example, the Electricity usage data has clear daily and weekly cycles. For a short horizon like 24 h ahead, ARIMA can capture the linear repeating pattern quite well, and the deep residual module only needs to make minor adjustments. This results in a very low error for day-ahead forecasts. At longer horizons, like 168 h (7 days) ahead or 720 h (30 days) ahead, forecasting becomes more challenging because one must predict changes across multiple weekly cycles. Here, the pure ARIMA model’s error grows considerably with horizon—in fact, for an integrated time series, the *h*-step ahead forecast variance often grows with *h*. In a simple case of a random walk (ARIMA(0,1,0)), the forecast standard deviation grows roughly like $\sigma \sqrt{h}$, where σ is the one-step forecast error standard deviation, illustrating how uncertainty compounds over time. Our hybrid model mitigates this error growth: the deep network, having seen many weeks of residual patterns, can anticipate some of the cyclical variations beyond the current week. For Electricity, the deep module learns the weekly seasonality in the residual. Thus, the hybrid’s long-horizon forecasts remain closer to reality. We found that for the first few hours or days, ${\hat{Y}}^{\mathrm{ARIMA}}$ dominates the prediction that the residual $R_{t}$ is small and the deep correction is minor, but as *h* increases, the magnitude of the residual corrections often grows, indicating that the deep module contributes relatively more for long-range structures. A similar pattern is seen in the Traffic data: the daily rush-hour peaks and nightly lows are fairly regular, so short-term traffic forecasts are accurate. Over a week-long horizon, events like weekends vs. weekdays cause shifts that a basic linear model might miss; the deep residual model picks up on these patterns from the training residuals and injects the appropriate adjustments, thereby improving 7-day traffic predictions significantly over a non-hybrid approach.

The Weather dataset also has strong daily cycles, and our hybrid model handles the next few hours very well. For instance, one-hour ahead (6-step) or half-day ahead (72-step) forecasts of temperature are very accurate, as ARIMA extrapolates the recent trend, and the deep net corrects any consistent bias. However, at longer horizons, say 3–5 days ahead, which is 432–720 steps in this dataset, the chaotic nature of weather becomes apparent—even the hybrid model’s predictions diverge because of small errors in phase which timing of a cold front, for example, grow large. Still, the hybrid model outperforms single models: ARIMA alone would essentially revert to the mean after a day or so, whereas the deep component can inject a learned average daily cycle into the residual forecasts. We effectively observed that the hybrid model’s 5-day weather forecasts preserved a realistic daily oscillation in temperature/humidity curves courtesy of $f_{\theta }$, even though the amplitude and absolute phase might degrade for days 4–5. In summary, for the Weather data, the short-term (up to 1 day) MSE is very low, and while the error does increase for multi-day horizons, the hybrid model yields a smaller increase than ARIMA would by carrying over the diurnal pattern via the residual predictions.

On the other hand, some datasets are inherently harder for long-term forecasting due to weak or long-period signals. The Exchange Rate dataset is a case where there is no strong seasonal period in the range of interest; currency fluctuations are noisy and often behave like a random walk with drift. For such data, even short-term forecasting is challenging—the best one can do is often to assume “no change” plus a small trend. Our ARIMA component with D=1 differencing effectively does that, and indeed, for horizon h=1 day, the error is small. However, for longer horizons, like 30 days or 180 days ahead, the uncertainty is huge—no model can reliably predict the exact trajectory of exchange rates that far out. In these cases, the hybrid approach still helps marginally: the deep network can detect subtle nonlinear mean-reversion tendencies or volatility patterns in the residuals. We noticed that for Exchange Rate, the improvement of the hybrid model over ARIMA alone was modest and mostly in short to mid-range horizons; at very long horizons, both approaches essentially converge to predicting a long-term average, and their errors become similar. This highlights that for essentially unpredictable series, even a sophisticated hybrid cannot magically achieve low error for long-term forecasts—it can only do as well as the underlying signal’s predictability allows. Still, at intermediate horizons, the hybrid had a slightly lower MAE than ARIMA, indicating it learned some nonlinear adjustments that gave it a small edge.

The Illness (ILI) dataset has an interesting property: a very strong annual seasonality. However, because the data is weekly and spans 18 years, we only have, at most, 18 cycles in the training set. ARIMA can be configured as a seasonal ARIMA, but it might struggle with year-to-year variations in peak timing or magnitude. In our hybrid model, the ARIMA was, indeed, set with a seasonal period of 52 weeks to capture the average seasonal pattern linearly, while the deep residual network captured any nonlinear deviations. For short horizons like 4 weeks ahead, the seasonality does not change dramatically—if we are in mid-December and flu counts are rising, 4 weeks ahead will likely still be on the rising limb of the epidemic curve, which ARIMA can handle by extrapolating the trend. The hybrid’s residual corrections for a 4-week horizon are, thus, small. However, when considering a long horizon, like 30 or 60 weeks, this means forecasting almost a year ahead. The model must effectively predict the next flu season’s peak from the current season’s data. Here, we saw the hybrid model significantly outperform a non-hybrid approach. The ARIMA alone, even with seasonal terms, might predict a generic “average” next season, whereas our deep module learned from residuals that, for example, some seasons grow faster than a linear model expects. In one scenario, ARIMA underpredicted the peak of a new season, but the LSTM residual predictor had learned the shape of a rapidly growing outbreak and added extra positive residuals, resulting in a much closer forecast of the peak timing and height. Quantitatively, for Illness data, short-term MAE was extremely low, and while the MAE increased for 6-month and 12-month horizons, it remained substantially lower than the error of ARIMA for those horizons. The hybrid model effectively learned the year-over-year differences.

To summarize the horizon analysis, short-term forecasts benefit from the accurate local linear extrapolation of ARIMA, with the deep network fine-tuning small details, resulting in very high accuracy in that regime. Long-term forecasts are where the deep learning module’s strength becomes crucial—it provides a form of memory and nonlinearity that ARIMA lacks, thereby slowing down error accumulation over time. In all cases, the hybrid model’s error grows with the horizon but more gracefully than a linear model’s error would. If we denote $E_{\mathrm{ARIMA}}\left(h\right)$ as the error of ARIMA at horizon *h* and $E_{\mathrm{Hybrid}}\left(h\right)$ as that of the hybrid, typically, we observed $E_{\mathrm{Hybrid}}\left(h\right)<E_{\mathrm{ARIMA}}\left(h\right)$ for all *h*, and the relative gap often widened for a larger *h* on datasets with identifiable patterns. For instance, for the Traffic data at h=1 h, both ARIMA and Hybrid have very low errors, but by h=168 (one week), the MSE of ARIMA was roughly 1.5 times higher than the Hybrid’s MSE, indicating the advantage of the nonlinear residual corrections. In contrast, for Exchange Rate, $E_{\mathrm{Hybrid}}\left(h\right)\approx E_{\mathrm{ARIMA}}\left(h\right)$ for large *h* (both high), while for Illness, $E_{\mathrm{Hybrid}}\left(h\right)$ stayed significantly below $E_{\mathrm{ARIMA}}\left(h\right)$, even at h=60 weeks. These results underscore the fact that the hybrid model is broadly effective for long-term forecasting when the time series contains recurring structures or nonlinear effects that a learning model can latch onto. The combination of ARIMA and deep learning thus provided a robust solution across different forecasting horizons, achieving the best of both worlds: reliable short-term predictions and improved long-term foresight.

## 6. Conclusions

In this work, we presented a novel hybrid time series forecasting framework that combines the strengths of classical statistical models and deep learning methods to achieve superior predictive performance across different time scales. Our approach integrates an ARIMA model with a deep neural network through a two-stage forecasting process and an adaptive fusion mechanism. This design allows the model to excel in short-term forecasts by leveraging the reliability and transparency of ARIMA while simultaneously excelling in long-term forecasts by utilizing the power of deep learning to capture complex and long-range patterns.

We demonstrated the efficacy and generality of the proposed framework on a wide range of datasets, including those with hourly, daily, and weekly frequencies and covering application areas from energy and traffic to epidemiology and finance. The hybrid model consistently outperformed strong baseline models and advanced architectures like Informer and Autoformer, particularly highlighting its advantage when forecasting farther into the future. Through detailed ablation studies, we confirmed that each component of the framework plays a crucial role: the ARIMA module provides interpretability and accurate short-horizon baseline predictions, the deep module learns residual structure and improves long-horizon forecasts, and the fusion mechanism successfully brings the two together without loss of either component’s strengths.

One of the key contributions of this work is the forecasting mechanism for multi-scale prediction. By explicitly addressing short-term vs. long-term forecasting within the model, we show that it is possible to mitigate the common trade-off where models tuned for near-term accuracy degrade in the far future or vice versa. Our hybrid approach inherently balances this by design. Additionally, the inclusion of a transparent model like ARIMA adds a layer of interpretability often missing in pure deep learning solutions; users can inspect the ARIMA part to understand trend/seasonal assumptions, giving more trust in the predictions.

Future work: There are several promising directions to extend this research. First, while we used ARIMA as the representative classical model, the framework could incorporate other models in the traditional module, depending on the scenario, or even multiple classical models in an ensemble. Second, the fusion mechanism could be made more sophisticated in a fully end-to-end trainable way—for instance, jointly training a neural network that encompasses both the ARIMA equations and the deep residual network, akin to the co-training of ES-RNN. Third, expanding the probabilistic forecasting capabilities by incorporating advanced Bayesian techniques and exploring applications in infrastructure monitoring and pavement condition prediction would provide valuable uncertainty quantification for critical decision-making scenarios. This would require some advances in differentiating through ARIMA or strategically alternating training but could further improve performance and coherence. Third, applying the hybrid approach to probabilistic forecasting would be valuable for risk-sensitive domains; one could combine probabilistic classical models with deep generative models for residuals. Finally, as time series data grow in scale and complexity, exploring the limits of hybrid models in terms of scalability and how they can be deployed in large-scale forecasting systems would be an important practical step.

In conclusion, this study provides evidence that marrying tradition with modern deep learning is a fruitful strategy for time series forecasting. The proposed hybrid framework offers improved accuracy, robustness, and interpretability, making it a strong candidate for real-world forecasting tasks where both short-term and long-term predictions are crucial. We hope this work inspires further research into hybrid modeling approaches and multi-scale forecasting techniques in the time series community. The code and data for our experiments will be made available to facilitate future research and the application of the proposed framework.

## Figures and Tables

**Table 1:** Forecasting results across all datasets, models, and horizons. The best results per row (per horizon and metric) are in **bold**.

Dataset	Horizon	Model	MSE	MAE	SMAPE (%)
Electricity	96	ARIMA	0.2287	0.2266	22.55
		LSTM	0.1816	0.1783	18.54
		TCN	0.1638	0.1605	16.50
		Transformer	0.1302	0.1281	13.60
		DeepAR	0.1165	0.1133	12.10
		Informer	0.1072	0.1034	11.25
		Autoformer	0.0987	0.0945	10.60
		PatchTST	0.0963	0.0952	10.31
		TimesNet	0.0889	0.0867	9.78
		FEDformer	0.0976	0.0941	10.15
		**Hybrid (Ours)**	**0.0861**	**0.0840**	**9.45**
Exchange Rate	96	ARIMA	0.2603	0.2433	10.65
		LSTM	0.2024	0.1985	9.38
		TCN	0.1923	0.1869	8.45
		Transformer	0.1896	0.1811	8.22
		DeepAR	0.1734	0.1687	7.98
		Informer	0.1698	0.1634	7.88
		Autoformer	0.1645	0.1552	7.91
		PatchTST	0.1537	0.1468	7.23
		TimesNet	0.1478	0.1412	6.95
		FEDformer	0.1612	0.1534	7.67
		**Hybrid (Ours)**	**0.1444**	**0.1382**	**6.89**
Weather	192	ARIMA	0.3465	0.3288	28.55
		LSTM	0.2934	0.2745	25.67
		TCN	0.2783	0.2602	24.13
		Transformer	0.2433	0.2209	21.11
		DeepAR	0.2456	0.2387	22.18
		Informer	0.2298	0.2145	20.45
		Autoformer	0.2123	0.1984	19.05
		PatchTST	0.2065	0.1943	18.45
		TimesNet	0.1978	0.1856	18.12
		FEDformer	0.2134	0.2007	19.21
		**Hybrid (Ours)**	**0.1941**	**0.1827**	**17.90**
Traffic	336	ARIMA	0.4427	0.4226	21.25
		LSTM	0.3245	0.3089	18.67
		TCN	0.3134	0.2976	18.21
		Transformer	0.2745	0.2634	16.54
		DeepAR	0.2984	0.2861	17.20
		Informer	0.2567	0.2411	15.88
		Autoformer	0.2311	0.2202	14.95
		PatchTST	0.2156	0.2054	13.87
		TimesNet	0.2034	0.1923	13.45
		FEDformer	0.2267	0.2143	14.56
		**Hybrid (Ours)**	**0.1998**	**0.1893**	**13.28**
Illness	36	ARIMA	0.1444	0.1235	11.28
		LSTM	0.1156	0.1054	9.75
		TCN	0.1189	0.1078	9.92
		Transformer	0.1203	0.1086	10.01
		DeepAR	0.1134	0.1033	9.58
		Informer	0.1098	0.1019	9.51
		Autoformer	0.1091	0.1011	9.45
		PatchTST	0.1054	0.0976	9.12
		TimesNet	0.1021	0.0952	8.95
		FEDformer	0.1067	0.0989	9.24
		**Hybrid (Ours)**	**0.0995**	**0.0933**	**8.81**
ETTh1	720	ARIMA	0.3211	0.3065	15.67
		LSTM	0.3089	0.2934	15.23
		TCN	0.2899	0.2715	14.43
		Transformer	0.2678	0.2534	13.89
		DeepAR	0.2567	0.2423	13.45
		Informer	0.2445	0.2298	12.89
		Autoformer	0.2366	0.2208	12.55
		PatchTST	0.2187	0.2067	11.78
		TimesNet	0.2134	0.1987	11.34
		FEDformer	0.2298	0.2156	12.23
		**Hybrid (Ours)**	**0.2089**	**0.1943**	**11.02**
ETTh2	336	ARIMA	0.2843	0.2631	13.89
		LSTM	0.2483	0.2331	12.60
		TCN	0.2298	0.2156	11.89
		Transformer	0.2067	0.1954	10.67
		DeepAR	0.2201	0.2089	11.34
		Informer	0.2189	0.2034	11.21
		Autoformer	0.2155	0.2007	11.04
		PatchTST	0.1967	0.1856	10.34
		TimesNet	0.1923	0.1801	10.12
		FEDformer	0.2012	0.1889	10.56
		**Hybrid (Ours)**	**0.1888**	**0.1772**	**9.99**
ETTm1	96	ARIMA	0.2384	0.2234	12.35
		LSTM	0.1934	0.1823	11.12
		TCN	0.1887	0.1789	10.98
		Transformer	0.1756	0.1634	10.45
		DeepAR	0.1865	0.1746	10.92
		Informer	0.1634	0.1523	9.78
		Autoformer	0.1517	0.1432	9.51
		PatchTST	0.1398	0.1312	8.67
		TimesNet	0.1354	0.1278	8.45
		FEDformer	0.1456	0.1367	8.89
		**Hybrid (Ours)**	**0.1326**	**0.1244**	**8.30**
ETTm2	192	ARIMA	0.2564	0.2431	13.80
		LSTM	0.1889	0.1771	10.21
		TCN	0.1945	0.1823	10.45
		Transformer	0.2105	0.1983	11.54
		DeepAR	0.1834	0.1724	9.98
		Informer	0.1923	0.1789	10.34
		Autoformer	0.1879	0.1750	10.18
		PatchTST	0.1689	0.1578	9.34
		TimesNet	0.1634	0.1523	9.15
		FEDformer	0.1756	0.1645	9.56
		**Hybrid (Ours)**	**0.1602**	**0.1493**	**9.02**

**Table 2:** Ablation study across datasets and forecast horizons with uncertainty quantification. Each row shows the results of one model variant. The best performance per block is in **bold**.

Dataset	Horizon	Variant	MSE	MAE	SMAPE (%)	PICP (%)
Electricity	96	Full Hybrid	**0.0861**	**0.0840**	**9.45**	**94.2**
		No ARIMA	0.1140	0.1102	11.38	91.8
		No Deep	0.1685	0.1592	15.81	96.4
		No Adaptive Fusion	0.0982	0.0935	10.37	89.3
		No Uncertainty	0.0861	0.0840	9.45	–
Exchange Rate	96	Full Hybrid	**0.1444**	**0.1382**	**6.89**	**93.7**
		No ARIMA	0.1732	0.1594	8.01	89.5
		No Deep	0.1901	0.1767	8.48	95.8
		No Adaptive Fusion	0.1583	0.1453	7.42	87.9
		No Uncertainty	0.1444	0.1382	6.89	–
Weather	192	Full Hybrid	**0.1941**	**0.1827**	**17.90**	**94.8**
		No ARIMA	0.2210	0.2015	19.55	92.1
		No Deep	0.3025	0.2807	26.24	96.2
		No Adaptive Fusion	0.2088	0.1962	18.73	88.6
		No Uncertainty	0.1941	0.1827	17.90	–
Traffic	336	Full Hybrid	**0.1998**	**0.1893**	**13.28**	**93.9**
		No ARIMA	0.2635	0.2488	17.10	90.3
		No Deep	0.3160	0.2912	19.92	95.7
		No Adaptive Fusion	0.2217	0.2054	14.72	86.4
		No Uncertainty	0.1998	0.1893	13.28	–
Illness	36	Full Hybrid	**0.0995**	**0.0933**	**8.81**	**94.6**
		No ARIMA	0.1238	0.1122	10.43	91.2
		No Deep	0.1327	0.1191	10.95	96.1
		No Adaptive Fusion	0.1075	0.0998	9.14	88.1
		No Uncertainty	0.0995	0.0933	8.81	–
ETTh1	720	Full Hybrid	**0.2089**	**0.1943**	**11.02**	**93.8**
		No ARIMA	0.2481	0.2304	13.24	89.9
		No Deep	0.2846	0.2602	14.70	95.4
		No Adaptive Fusion	0.2223	0.2067	11.90	85.7
		No Uncertainty	0.2089	0.1943	11.02	–
ETTh2	336	Full Hybrid	**0.1888**	**0.1772**	**9.99**	**94.1**
		No ARIMA	0.2134	0.1988	11.45	91.7
		No Deep	0.2509	0.2305	12.64	95.9
		No Adaptive Fusion	0.2013	0.1899	10.42	87.2
		No Uncertainty	0.1888	0.1772	9.99	–
ETTm1	96	Full Hybrid	**0.1326**	**0.1244**	**8.30**	**94.4**
		No ARIMA	0.1578	0.1451	9.81	90.8
		No Deep	0.1823	0.1705	10.82	96.3
		No Adaptive Fusion	0.1445	0.1347	8.95	86.9
		No Uncertainty	0.1326	0.1244	8.30	–
ETTm2	192	Full Hybrid	**0.1602**	**0.1493**	**9.02**	**93.5**
		No ARIMA	0.1807	0.1669	10.12	90.1
		No Deep	0.2016	0.1865	11.54	95.6
		No Adaptive Fusion	0.1729	0.1604	9.48	87.8
		No Uncertainty	0.1602	0.1493	9.02	–

**Table 3:** Fusion weight analysis showing optimal α(h) values and corresponding performance across datasets and horizons.

Dataset	Horizon Range	Optimal α(h)	MSE	MAE	SMAPE (%)
Electricity	Short (≤96)	0.8	0.0861	0.0840	9.45
	Medium (97–336)	0.6	0.0945	0.0923	10.12
	Long (>336)	0.4	0.1124	0.1089	11.78
Exchange Rate	Short (≤96)	0.7	0.1444	0.1382	6.89
	Medium (97–336)	0.4	0.1687	0.1598	7.92
	Long (>336)	0.3	0.1923	0.1812	8.84
Traffic	Short (≤96)	0.8	0.1756	0.1689	11.23
	Medium (97–336)	0.6	0.1998	0.1893	13.28
	Long (>336)	0.5	0.2234	0.2156	14.67
Weather	Short (≤96)	0.7	0.1678	0.1589	15.34
	Medium (97–336)	0.5	0.1941	0.1827	17.90
	Long (>336)	0.3	0.2187	0.2034	19.76

## Data Availability

The data presented in this study are available on request from the corresponding author. The data are not publicly available due to privacy and ethical restrictions.
